# Type I Interferon Responses by HIV-1 Infection: Association with Disease Progression and Control

**DOI:** 10.3389/fimmu.2017.01823

**Published:** 2018-01-15

**Authors:** Andrew Soper, Izumi Kimura, Shumpei Nagaoka, Yoriyuki Konno, Keisuke Yamamoto, Yoshio Koyanagi, Kei Sato

**Affiliations:** ^1^Laboratory of Systems Virology, Department of Biosystems Science, Institute for Frontier Life and Medical Sciences, Kyoto University, Kyoto, Japan; ^2^Graduate School of Medicine, Kyoto University, Kyoto, Japan; ^3^Graduate School of Pharmaceutical Sciences, Kyoto University, Kyoto, Japan; ^4^Graduate School of Biostudies, Kyoto University, Kyoto, Japan; ^5^CREST, Japan Science and Technology Agency, Kawaguchi, Japan

**Keywords:** type I interferon, human immunodeficiency virus type 1, innate immunity, intrinsic immunity, interferon-stimulated gene, restriction factor, humanized mouse

## Abstract

Human immunodeficiency virus type 1 (HIV-1) is the causative agent of acquired immunodeficiency syndrome and its infection leads to the onset of several disorders such as the depletion of peripheral CD4^+^ T cells and immune activation. HIV-1 is recognized by innate immune sensors that then trigger the production of type I interferons (IFN-Is). IFN-Is are well-known cytokines eliciting broad anti-viral effects by inducing the expression of anti-viral genes called interferon-stimulated genes (ISGs). Extensive *in vitro* studies using cell culture systems have elucidated that certain ISGs such as APOBEC3G, tetherin, SAM domain and HD domain-containing protein 1, MX dynamin-like GTPase 2, guanylate-binding protein 5, and schlafen 11 exert robust anti-HIV-1 activity, suggesting that IFN-I responses triggered by HIV-1 infection are detrimental for viral replication and spread. However, recent studies using animal models have demonstrated that at both the acute and chronic phase of infection, the role of IFN-Is produced by HIV or SIV infection in viral replication, spread, and pathogenesis, may not be that straightforward. In this review, we describe the pluses and minuses of HIV-1 infection stimulated IFN-I responses on viral replication and pathogenesis, and further discuss the possibility for therapeutic approaches.

## Human Immunodeficiency Virus Type 1 (HIV-1) Recognition for Type I Interferon (IFN-I) Production

**Figure 1:**
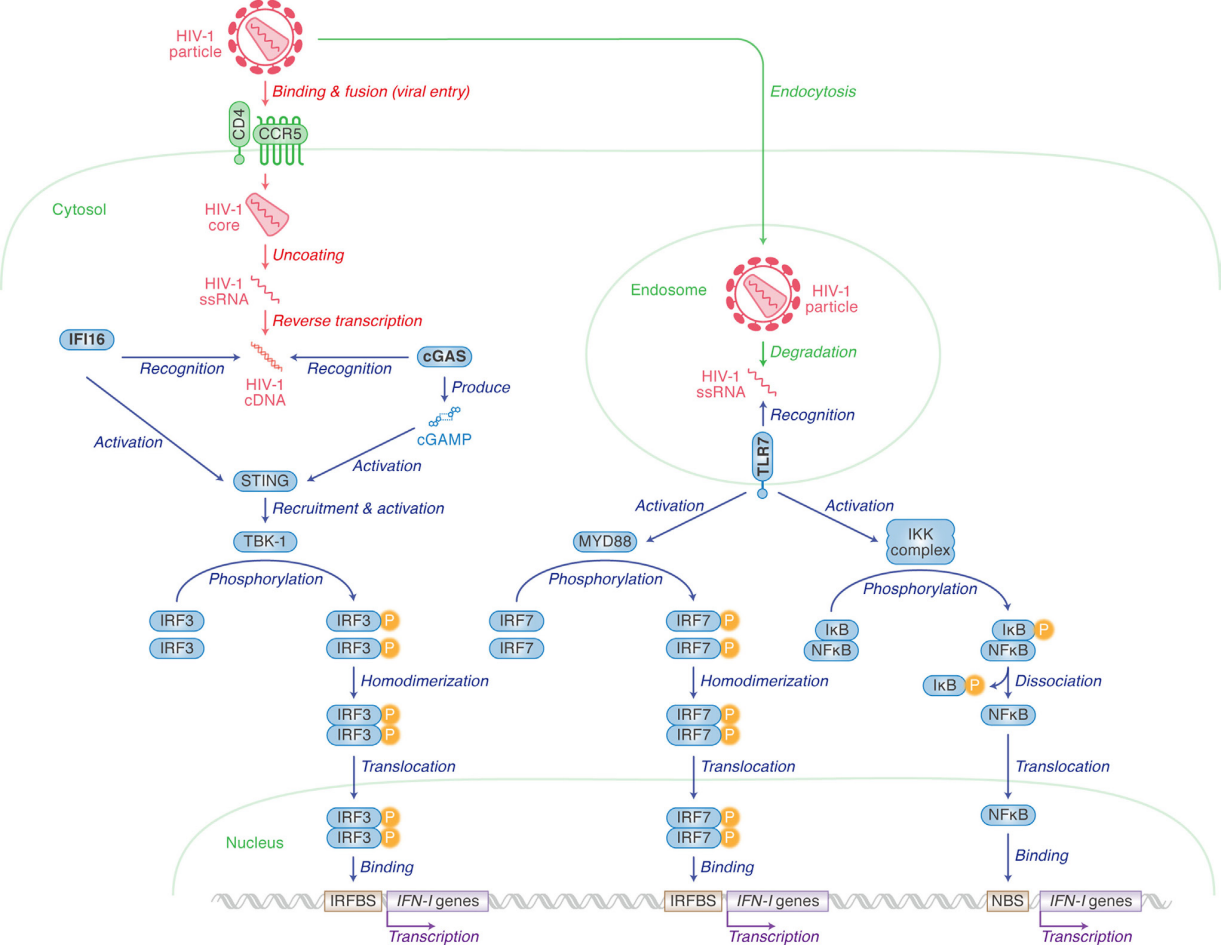
Pattern recognition receptors (PRRs) for human immunodeficiency virus type 1 (HIV-1) recognition and the following pathway for triggering type I interferon (IFN-I) expression. Cellular actions are indicated in green (in italic) with arrows, and viral replication steps are indicated in red (in italic) with arrows. Cellular actions triggered by PRRs [IFN-γ inducible protein 16 (IFI16), cyclic GMP-AMP synthase (cGAS) and toll-like receptor 7 (TLR7)] are indicated in blue (in italic) with arrows. Cellular organelle, viral components, and sensor-related molecules are indicated in green, red, and blue, respectively. “P” with yellow circle indicates phosphorylation. The detail of each step is described in the main text.

Human immunodeficiency virus type 1 infection in humans induces innate immune responses mediated mainly by IFN-I, including IFN-α and IFN-β, and the roles of IFN-I in responding to HIV-1 infection have been reviewed extensively (1–3). Upon HIV-1 infection into human immune cells, pattern recognition receptors (PRRs) and cytosolic sensors are involved in the sensing of viral cDNA or RNA, respectively. After HIV-1 infects human cells, cDNA is synthesized by RNA reverse transcription. cDNA is then recognized by either IFN-γ inducible protein 16 (IFI16) or cyclic GMP-AMP (cGAMP) synthase (cGAS) (4–9). cGAS especially recognizes cDNA and subsequently produces cGAMP. IFI16 and cGAMP both activate stimulator of interferon gene (STING; also known as transmembrane protein 173). Activated STING in turn recruits and activates TANK binding kinase 1 which phosphorylates IFN regulatory factor 3 (IRF3). Finally, IFN-I is produced by IRF3 in the pathways highlighted on the left of Figure 1 (10–17).

IFI16 is expressed in epithelial cells, fibroblasts, and endothelial cells (4), as well as cells from hematopoietic lineages such as macrophages (5) and CD4^+^ T cells (6). Contrastingly, the cGAS-STING pathway is not present in T cells (6), but does play an important role in IFN-I production in myeloid lineages including macrophages (7) and monocyte-derived dendritic cells (MDDCs) (8, 9). As CD4^+^ T cells are more permissive to HIV-1 infection and replication than macrophages and MDDCs, this can probably be explained by the lack of cGAS expression in CD4^+^ T cells (5, 6).

HIV-1 single-stranded RNA can also be sensed by toll-like receptor 7 (TLR7), a PRR, when viruses are enclosed by endosomes (10, 11). Unlike IFI16 and cGAS, plasmacytoid dendritic cells (pDCs) express high levels of TLR7 (12, 13). TLR7 mediates another cascade ultimately resulting in either IRF7 homodimers translocating to the nucleus to bind to IRFBS, or the freeing of NFκB to activate the transcription of *IFN-I* genes *via* binding to the NFκB binding site (14).

## IFN-Stimulating Genes (ISGs): Effector Molecules Exhibiting Anti-Viral Effects

**Figure 2:**
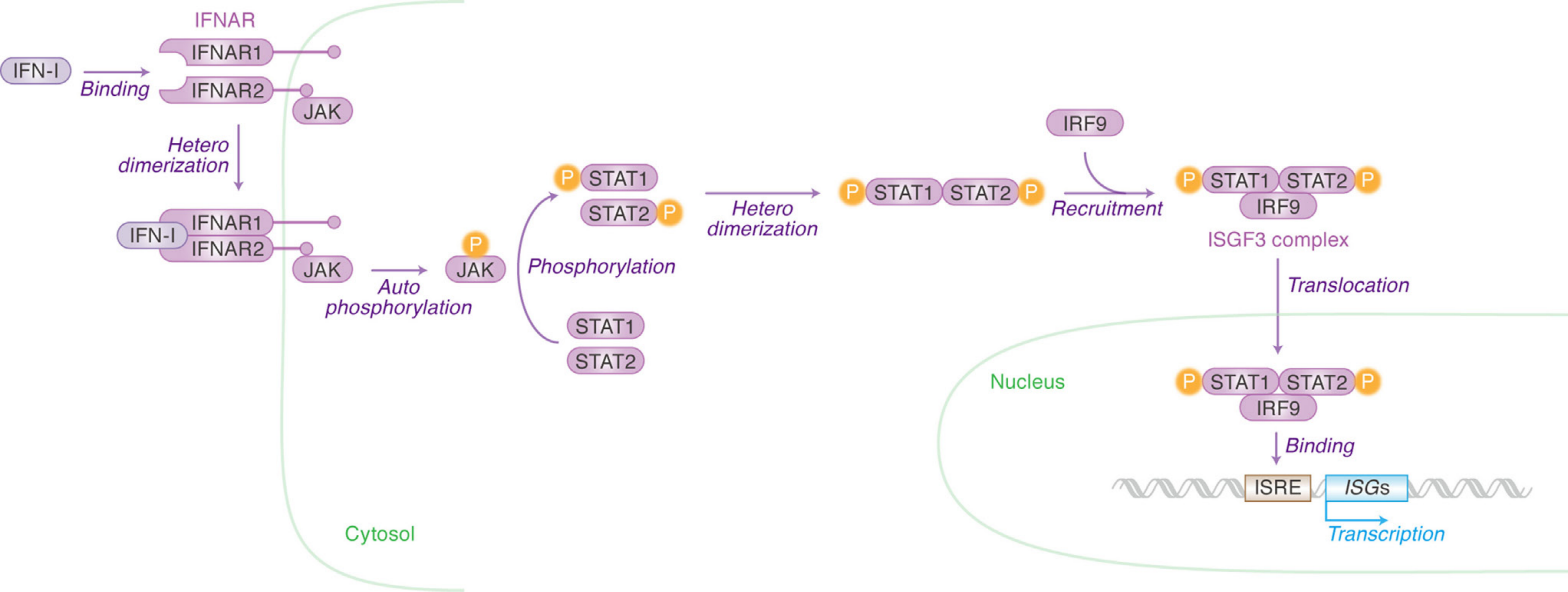
Type I interferons (IFN-I)-initiated signaling pathway leading to interferon-stimulated gene (ISG) expression. Cellular actions triggered by IFN-I [Janus kinase (JAK)/signal transducer and activator of transcription (STAT) pathway] are indicated in purple (in italic) with arrows. Cellular organelle and the cellular molecules related to JAK/STAT pathway are indicated in green and purple, respectively. The detail of each step is described in the main text. “P” with yellow circle indicates phosphorylation.

Once IFN-I is produced, this protein binds to its receptor molecule that is expressed on the cell surface. IFN-I receptor (IFNAR) consists of two independent proteins, IFNAR1 (IFN-α/β receptor α chain) and IFNAR2 (IFN-α/β receptor β chain) (Figure 2). Binding of the ligand IFN-I to the IFN-I receptor induces the heterodimerization of IFNAR1 and IFNAR2, which leads to the autophosphorylation of Janus kinase (JAK) (Figure 2). The phosphorylated JAK then induces the heterodimerization of signal transducer and activator of transcription 1 (STAT1) and STAT2 *via* phosphorylation (Figure 2). This cascade is known as the JAK/STAT pathway. The STAT1–STAT2 heterodimer recruits IFN regulatory factor 9 and forms the IFN-stimulated gene factor 3 (ISGF3) complex. After the entry of ISGF3 complex into the nucleus, this complex binds to the IFN-stimulated response element located in the promoter region of ISGs and initiates their transcription (Figure 2) (15). There are 17 subtypes of IFN-Is (16), and there have now been over 300 ISGs identified. In humans, however, is it not known in which tissues the different INF-α isoforms are expressed upon viral infection nor which cells express them. This is an intriguing issue and will no doubt be revealed in future investigations using techniques such as next generation sequencing.

## Restriction Factors (RFs): ISGs Potently Control HIV-1 Replication

**Figure 3:**
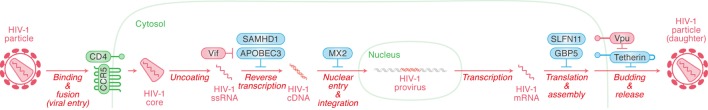
Restriction factors (RFs) controlling human immunodeficiency virus type 1 (HIV-1) replication and viral antagonists. Viral replication steps are indicated in red (in italic) with arrows. The RFs inhibiting viral replication at respective step are indicated in cyan, while the viral accessory proteins counteracting the action of certain RFs are indicated in red. Cellular organelle and viral components are indicated in green and red, respectively. The detail of each step is described in the main text.

Type I interferon treatment efficiently suppresses HIV-1 replication in *in vitro* cell cultures (17), meaning that certain ISGs potently control HIV-1 replication. Among the more than 300 known ISGs, certain ones are known to exhibit robust anti-HIV-1 activity and these ISGs are referred to as “intrinsic immunity” or “RFs.” Although the types of RFs appear numerous, the most well studied to date include SAM domain and HD domain-containing protein 1 (SAMHD1) and apolipoprotein B mRNA editing enzyme catalytic-like 3 (APOBEC3) (targeting HIV-1 reverse transcription), MX dynamin-like GTPase 2 (MX2) (targeting nuclear entry), schlafen 11 (SLFN11) (targeting transcription), guanylate-binding protein 5 (GBP5) (targeting post-translational modification), and tetherin (targeting release) (Figure 3). In this section, we briefly summarize the restriction mechanisms employed by RFs that inhibit HIV-1 replication at multiple stages.

### SAM Domain and HD Domain-Containing Protein 1

During the process of HIV-1 reverse transcription, viral reverse transcriptase requires deoxynucleoside triphosphates (dNTPs) as a substrate for the synthesis of viral cDNA (18, 19). SAMHD1 is a cytosolic enzyme with phosphohydrolase activity that enzymatically degrades (“hydrolyzes”) dNTPs (18–20). Deoxyguanosine triphosphate in particular, binds to the allosteric site of SAMHD1 and activates SAMHD1’s hydrolytic activity (18).

SAM domain and HD domain-containing protein 1 is expressed in peripheral CD4^+^ leukocytes including myeloid cells [e.g., macrophages and dendritic cells (DCs)] and CD4^+^ T cells (19). The experiments in *in vitro* cell cultures demonstrate that SAMHD1 restricts HIV-1 infection in non-dividing cells such as macrophages (plus phorbol 12-myristate 13-acetate-stimulated macrophage-like THP-1 cell line), DCs, and resting CD4^+^ T cells by degrading dNTPs (18, 19).

In comparison with dividing (i.e., cycling and activated/proliferating) cells, the level of intracellular dNTP is much lower in non-dividing cells (21). Previous studies have suggested that SAMHD1 plays a crucial role in maintaining a low pool of cellular dNTPs in non-dividing cells, including resting CD4^+^ T cells, which may reduce the risk of retroviral insult without disrupting homeostasis in the non-dividing cell environment (21, 22). In dividing cells, including activated CD4^+^ T cells, SAMHD1 is post-transcriptionally inactivated: cyclin-dependent kinases 1 (CDK1) and CDK2 phosphorylate the threonine residue at position 592 of SAMHD1 (23). This phosphorylation impairs SAMHD1’s hydrolyzing activity and results in the loss of its anti-HIV-1 activity (23). CDKs, including CDK1 and CDK2, are key regulators of the cell cycle that activate cyclins during cell division (24). As dividing cells require a greater pool of dNTPs, SAMHD1’s enzymatic activity is inhibited by CDK1/2-mediated phosphorylation (25).

To overcome SAMHD1-mediated restriction, an accessory protein of human lentiviruses, viral protein X (Vpx), degrades SAMHD1 *via* the ubiquitin/proteasome-dependent pathway (22). The Q76A mutation in Vpx results in the loss of SAMHD1 degradation ability suggesting that the glutamine at position 76 is critical (22). Importantly, the *vpx* gene is not encoded by HIV-1 but HIV-2, another human lentivirus and causative agent of acquired immunodeficiency syndrome (AIDS) (26).

Human immunodeficiency virus type 1 and HIV-2 are evolutionarily and phylogenetically distinct, and more intriguingly, Etienne et al. have shown evidence indicating that the lineage of primate lentiviruses, including HIV-1, lost the *vpx* gene during viral evolution (27). These observations raise an incongruous insight: although RFs such as APOBEC3 and tetherin (see below) can be degraded and antagonized by HIV-1 accessory proteins, HIV-1 does not possess any counterparts to counteract SAMHD1. Additionally, HIV-1 is able to replicate in macrophages that express SAMHD1 in its anti-viral state. Moreover, HIV-1 is more pathogenic than HIV-2 in spite of the absence of anti-SAMHD1 factor(s) (26). These insights may imply that SAMHD1 is not critical for the restriction of HIV-1 replication. In this regard, a previous paper has revealed that the concentration of dNTP required for the reverse transcriptase of HIV-1 is clearly lower than that of HIV-2, and HIV-1 can efficiently reverse transcribe a single strand template with a lower level of dNTPs (21). Therefore, HIV-1 may have evolved to overcome SAMHD1-mediated anti-viral activity by decreasing the requirement for a high-dNTP concentration.

In addition to the phosphohydrolase activity, SAMHD1 possesses ribonuclease (RNase) activity. Ryoo et al. have reported that RNase activity but not phosphohydrolase is required for exhibiting an anti-HIV-1 effect (28). This is shown with the D137N mutant of SAMHD1, which possesses RNase activity but specifically loses phosphohydrolase activity and is still able to restrict HIV-1 infection. In contrast, the Q548A mutant of SAMHD1 that loses RNase activity but maintains phosphohydrolase activity is ineffective at restricting HIV-1 (28). Moreover, the phosphorylation of SAMHD1 at T592 negatively regulates its RNase activity in cells and impedes HIV-1 restriction (28), suggesting that the RNase activity of SAMHD1 is responsible for preventing HIV-1 infection by directly degrading viral RNA (28).

### Apolipoprotein B mRNA Editing Enzyme Catalytic-Like 3

Apolipoprotein B mRNA editing enzyme catalytic-like 3 family proteins are cellular cytidine/cytosine deaminases and the human genome encodes seven *APOBEC3* genes: *APOBEC3A, B, C, D, F, G*, and *H*. Some APOBEC3 family proteins, particularly APOBEC3D, APOBEC3F, APOBEC3G, and certain haplotypes of APOBEC3H (see below), are incorporated into released viral particles and enzymatically remove the amino group (-NH_2_) of the cytosine residue in the minus-stranded viral DNA during viral reverse transcription. This deamination converts cytosine to uracil, which results in guanine (G) to adenine (A) substitution in the plus-stranded viral DNA (this step is usually referred to as “APOBEC3-mediated G-to-A mutation”). The APOBEC3-mediated G-to-A mutations can result in the insertion of premature termination mutations [e.g., if TGG codon is converted to TGA codon by APOGEC3, the codon encoding tryptophan (TGG) is converted to a stop codon (TGA)]. Also, multiple APOBEC3-mediated G-to-A mutations can lead to the accumulation of non-synonymous mutations, which may produce defective viral proteins.

To overcome APOBEC3-mediated anti-viral action, an accessory protein of HIV-1, viral infectivity factor (Vif), degrades anti-viral APOBEC3 proteins in virus-producing cells *via* the ubiquitin/proteasome-dependent pathway. The relationship between APOBEC3 and Vif has been well studied and reviewed previously [e.g., Ref. (29, 30)].

To elucidate the roles of endogenous APOBEC3 proteins in HIV-1 infection *in vivo*, hematopoietic stem cell (HSC)-transplanted “humanized” mouse models have been utilized. First, *vif-*deficient HIV-1 was incapable of replicating in humanized mice, indicating that Vif is a prerequisite for HIV-1 infection and replication *in vivo* (31). Also, some proviral DNA in infected humanized mice exhibited G-to-A hypermutations, further suggesting that endogenous APOBEC3 protein(s) potently exhibit anti-HIV-1 activity *in vivo* (31).

Secondly, to elucidate which endogenous APOBEC3 protein(s) crucially affect HIV-1 replication *in vivo*, two *vif* mutants have been utilized: one is designated “4A,” which is unable to antagonize APOBEC3D and APOBEC3F, while the other is designated “5A,” which is unable to antagonize APOBEC3G (32). As the replication efficacy of both 4A and 5A HIV-1 were significantly lower than that of wild-type (i.e., *vif-*proficient) HIV-1, endogenous APOBEC3D, APOBEC3F, and APOBEC3G are deemed to be potent intrinsic RFs in humanized mice (32). On the other hand, it is intriguing that the viral RNA in the plasma of humanized mice infected with 4A HIV-1 had greater diversity compared with 5A and wild-type HIV-1 sequences (32). This observation suggests that the G-to-A mutation caused by APOBEC3D and APOBEC3F potently contributes toward viral diversification. In this regard, APOBEC3G prefers the GG-to-AG mutation, while APOBEC3D and APOBEC3F prefer a GA-to-AA mutation (33–35). Also, an experimental-mathematical analysis has suggested that APOBEC3G-mediated substitution easily results in nonsense mutations (mainly because “TGG,” a codon encoding tryptophan, is converted to “TAG,” a stop codon), while the G-to-A mutations mediated by APOBEC3D and APOBEC3F (i.e., GA-to-AA mutation) lead only to missense mutations (33). Therefore, three endogenous APOBEC3 proteins, APOBEC3D, APOBEC3F, and APOBEC3G, possess the ability to suppress HIV-1 replication *in vivo*, while, at the same time, APOBEC3D and APOBEC3F may promote viral diversification.

Third, Nakano et al. have recently addressed the anti-viral effect of APOBEC3H *in vivo* (36). There are seven haplotypes within human *APOBEC3H* genes and APOBEC3H can be categorized based on the protein expression status of three phenotypes: stable (haplotypes II, V, and VII), intermediate (haplotype I), and unstable (haplotypes III, IV, and VI) (37–39). From the retrovirological point of view, only stable APOBEC3H exhibits anti-HIV-1 activity (37–39). Interestingly, although almost all of the naturally occurring HIV-1 Vif proteins can antagonize anti-viral APOBEC3 proteins including APOBEC3D, APOBEC3F, and APOBEC3G, certain Vif proteins are incapable of counteracting stable (i.e., anti-viral) APOBEC3H and are called “hypo” Vif (37). On the other hand, the Vif proteins that can antagonize stable APOBEC3H are called “hyper” Vif (37). To investigate the impact of endogenous APOBEC3H *in vivo*, an “*in vivo* competition assay” was conducted: hyper and hypo HIV-1s were co-inoculated into humanized mice encoding stable or unstable APOBEC3H and the most efficiently replicating virus was determined by RT-PCR (36). In the humanized mice encoding stable APOBEC3H, hyper HIV-1 predominantly replicated, suggesting that Vif’s ability to antagonize stable APOBEC3H is a prerequisite when the host is expressing stable APOBEC3H (36). On the other hand, since the type of virus that efficiently replicated in the humanized mice encoding unstable APOBEC3H (i.e., “hyper” or “hypo”) was stochastic, the selection pressure mediated by unstable APOBEC3H is relaxed (36). Moreover, hyper HIV-1 has emerged in the mice encoding stable APOBEC3H, originally infected with hypo HIV-1 (36). Altogether, these findings suggest that stable variants of APOBEC3H impose selective pressure on HIV-1. More importantly, the expression levels of these *APOBEC3* genes are upregulated in the CD4^+^ T cells of humanized mice infected with HIV-1 (32, 36). As global transcriptome analyses have also indicated the upregulation of ISG expression levels (36), it can be said; IFN-I responses can be triggered by HIV-1 infection in humanized mice.

### MX Dynamin-Like GTPase 2

The human genome encodes two IFN-inducible MX dynamin-like guanosine triphosphate hydrolases (GTPases), MX1 and MX2 (also known as MXA and MXB, respectively), which are presumably created by gene duplication over the course of evolution (40, 41). In addition to MX2’s strong anti-HIV-1 activity (42–44), it has also been shown that MX1 can suppress a wide range of other pathogenic DNA and RNA viruses, not including HIV-1 (42, 44, 45).

It is well known that IFN-I stimulation strongly inhibits HIV-1 infection during the early stages of viral replication (i.e., from entry to integration process) (46). To determine the IFN-I-responsive RF(s) restricting HIV-1 replication, comparative gene expression profiling (i.e., mRNA microarray) was conducted using human cells in the presence and the absence of IFN-I, and identified MX2 as the determining factor (42, 44). Subsequent investigations revealed that MX2 participates in blocking HIV-1 infection after reverse transcription (42–44). Since MX2 overexpression reduces the levels of nuclear viral DNA (e.g., 2-LTR circles) and more efficiently suppresses HIV-1 infection in non-dividing cells when compared with dividing cells (42, 44), MX2 presumably inhibits nuclear import of the viral complex. Moreover, MX2-mediated anti-viral potency is dependent on the viral capsid protein, as N57S and G89V mutants in the HIV-1 capsid render resistance to MX2 (42). Furthermore, a previous study has suggested that MX2-mediated restriction can be overcome by the depletion of cyclophilin A (CYPA), a peptidylprolyl isomerase (officially designated PPIA), and the treatment of cyclosporin A, a compound inhibiting CYPA (43). These findings suggest that CYPA is required for MX2-mediated anti-viral activity. CYPA is a well-known interaction partner of the HIV-1 capsid protein [reviewed in Ref. (47)]. Therefore, it is plausible that MX2 is closely associated with the viral complex composed of HIV-1 capsid and lines of cellular proteins.

Guanosine triphosphate hydrolase activity is required for the anti-viral effect of MX1 (48). In contrast, the K131A and T151A mutants of MX2, which lose the ability of GTP binding and hydrolysis, respectively, still exhibit anti-HIV-1 activity that is comparable with wild-type MX2 (42), suggesting that the MX2’s enzymatic activity appears to be dispensable for its anti-viral effect. On the other hand, the deletion of the nuclear localization signal at the N-terminus of MX2 results in the loss of anti-viral activity (42). These observations suggest the importance of the nuclear localization signal for MX2 to exhibit anti-HIV-1 activity, although the functional importance of the subcellular localization of MX2 remains unclear.

### Schlafen 11

Schlafen 11 is also an ISG and potently inhibits HIV-1 production (49). Since SLFN11 overexpression suppresses viral protein expression but not viral transcription, this RF restricts viral replication at a post-transcriptional stage (49). Interestingly, the protein expression of codon-optimized HIV-1 Gag as well as GFP does not affect SLFN11 overexpression or knockdown. Moreover, SLFN11 binds to tRNA and impairs protein expression based on codon usage (49). Altogether, SLFN11 restricts HIV-1-biased translation in a codon-usage-dependent manner (49). Furthermore, a subsequent study has revealed that primate *SLFN11* genes are under evolutionarily positive selection pressure and commonly possess the ability to impair viral production regardless of the virus or host target (50). However, it remains unclear how SLFN11 influences HIV-1 replication *in vivo*.

### Guanylate-Binding Protein 5

Guanylate-binding protein 5 belongs to an IFN-inducible subfamily of GTPases with host defense activity against intracellular bacteria and parasites (51). Krapp et al. have recently demonstrated that GBP5 suppresses HIV-1 infectivity by interfering with *N*-linked glycosylation of the viral envelope glycoprotein (Env) (52). The cysteine residue at position 583 is critical for its anti-viral activity; however, catalytically inactive mutants still demonstrate anti-viral activity, suggesting GBP5 exhibits an anti-viral effect independent of its enzymatic activity (52).

Intriguingly, the nonsense mutations in the HIV-1 *vpu* gene (i.e., the deletion of initiation codon or the insertion of premature stop codons) increase Env expression and confer resistance to GBP5-mediated anti-viral activity (52). As described below, viral protein U (Vpu) is a crucial factor for the counteraction of tetherin-mediated restriction (53, 54). However, it should be noted that the initiation codons of the *vpu* gene in certain clinical HIV-1 isolates including HXB2 (55), BH8 (56), MAL (57), and Zr6 (58) are primarily deleted. Additionally, the expression of *GBP5* gene is upregulated by HIV-1 replication in infected individuals (52). Therefore, it might be plausible to assume that conferring resistance to GBP5 is important for HIV-1 dissemination in certain tissues or organs in infected individuals, and that there is a “trade-off” relationship between anti-tetherin activity (presence of Vpu) and GBP5 resistance (absence of Vpu).

### Tetherin

The observation that the HIV-1 accessory protein, Vpu, is required for the efficient release of HIV-1 particles depending on cell type indicated the existence of an RF counteracted by Vpu (59–64). In 2008, Neil et al. and Van Damme et al. identified tetherin (also known as bone marrow stromal antigen 2, CD317, and HM1.24) (53, 54). Tetherin is an IFN-I-inducible type II membrane protein that consists of an *N*-terminal cytoplasmic tail, a transmembrane domain, and an extracellular domain with a glycosylphosphatidylinositol (GPI) modification at the C-terminus (65). Due to GPI anchoring, tetherin is mainly localized in cholesterol-enriched lipid rafts (65), where HIV-1 viruses bud from, and retains budding virions on the plasma membrane of virus-producing cells (66). To antagonize the tetherin-mediated anti-viral action, Vpu downregulates tetherin from the surface of HIV-1-producing cells (54, 67). Vpu is a multifunctional type I transmembrane protein [reviewed in Ref. (68)] and sequesters tetherin molecules from the cell surface to endosomal compartments through transmembrane domain-mediated interaction (69–72). Additionally, the DSGXXS motif in the cytoplasmic tail of Vpu interacts with BTRC1 (beta-transducin repeat containing E3 ubiquitin protein ligase; also known as β-TrCP1 and Fbxw1), a subunit of E3 ubiquitin ligase. In this way, Vpu induces tetherin ubiquitination and enhances subcellular sorting of tetherin mediated by endosomal sorting complexes required for transport machinery into lysosomal compartments for degradation (72). The requirement of BTRC1 for tetherin antagonization, however, remains controversial.

To reveal the importance of Vpu in the dynamics of HIV-1 replication *in vivo*, Sato et al. (73) and Dave et al. (74) utilized HSC-transplanted humanized mouse models and demonstrated that Vpu strongly downregulates the expression level of tetherin on the surface of virus-producing cell *in vivo*. The replication kinetics of *vpu-*deficient HIV-1 during the early phase of infection is clearly lower than that of wild-type HIV-1 in humanized mice (73, 74), suggesting that Vpu augments HIV-1 replication during the acute phase of infection.

In addition to the tetherin’s ability to impair viral release, it can also be an inducer of NFκB activation (75, 76). The molecular mechanism of tetherin-mediated NFκB activation has been well investigated in *in vitro* cell cultures (75–77). However, the importance of NFκB signaling triggered by tetherin in HIV-1 replication *in vivo* remains unknown and needs to be addressed in future investigations.

## IFN-I Responses and Immunity Against HIV-1 Infection

Acquired immunodeficiency syndrome is one of many sexually transmitted diseases and HIV-1 infection is accomplished *via* mucosal transmission (78). Notably, transmitter/founder viruses that are transferred from infected patients to nascent individuals are apparently resistant to IFN-I-mediated anti-HIV-1 effects (79–81). These insights strongly suggest that RFs induced by IFN-I are involved in protecting infected individuals at many stages, from HIV-1 acquisition at the mucosal level (i.e., vagina and rectum), right through to limiting virus replication once infection has occurred. However, it remains unclear how transmitted/founder viruses exhibit such resistance to IFN-I (and presumably to the RFs induced by IFN-I).

The source of IFN-I in the acute phase of infection is thought to primarily be pDCs that reside in the mucosa (82, 83). In contrast, it is still unclear which cells are the primary sources of IFN-I during chronic infection. Almost all nucleated cells can produce IFN-Is in times of viral infection (84), pDCs being the largest producers (85, 86). IFN-Is can then act upon NK cells in an autocrine fashion (87–90) or else on macrophages (91). pDCs are found in the circulation but are also capable of dispersing into both lymphoid and most frequently, gut mucosal tissues (92–94). Similar to the other types of leukocytes, NK cells are activated by IFN-I and exhibit high cytotoxic activity (95). The NK cells activated by both IFN-α and TNF-α can suppress HIV-1 viral replication *via* the secretion of CCL3/4/5, IFN-γ, TNF-α, and GM-CSF (96–99). It is also known that IL-12 secreted by DCs and/or macrophages in combination with IFN-, stimulates NK cells to secrete higher amounts of IFN-γ (90).

As described above, the IFN-I responses induced by HIV-1 infection are assumed to contribute to the building up of an anti-viral environment in infected patients. However, it remains controversial as to whether or not IFN-I responses are beneficial for infected patients. For instance, with increased IFN-I production in pDCs, there is an increase in RANTES (regulated on activation, normal T cell expressed and secreted; also known as MIP-1α), a CCR5 ligand, aiding in the recruitment of further target cells, which probably contributes to enhanced viral expansion (82). Additionally, the IFN-α produced by pDCs is both capable of inhibiting the proliferation of bystander CD4^+^ T cells (100), and promoting the apoptosis of uninfected bystander CD4^+^ T cells residing in the lymphoid tissue of HIV-1-infected patients (101).

While IFN-β, a subtype of IFN-I, administered *via* the vagina was shown to protect against systemic infection of simian/HIV, a chimeric virus of SIV and HIV in rhesus macaque monkeys (102); the treatment of IFN-I for HIV-1-infected individuals was not successful [reviewed in Ref. (103)]. However, elite controllers, who are able to control HIV-1 infection without any treatment maintain higher pDC counts and IFN-α production compared with viremic patients and infected patients on combination anti-retroviral therapy (cART; previously called highly active anti-retroviral therapy) (104), suggesting that IFN-Is play pivotal roles in controlling HIV-1 infection in elite controllers. In consideration of why IFN-I treatment was not successful, most prior studies have used IFN-I subtype, IFN-α2, as standalone treatments, putative vaccine, or adjuvants for cART in patients (3). However, it has been recently suggested that IFN-α8 and IFN-α14, alternative types of IFN-I, may be better suited as these possess a higher affinity for the IFNAR and consequently result in a greater expression of certain RFs such as MX2, tetherin, and APOBEC3 (105, 106). The complicated effect of IFN-I responses subsequent to HIV-1 infection and recent observations in *in vivo* animal models are described in the following section.

## Effect of IFN-I on HIV-1 Infection *In Vivo*

Investigations using HSC-transplanted humanized mouse models have recently suggested that the initial burst of IFN-I is extremely important in controlling the acute phase of HIV-1 infection to limit reservoir size and disease course (105, 107). However, a sustained IFN-I response is detrimental as it contributes to increased systemic inflammation (108). It has also been shown in humanized mice that NK cells possess the ability to inhibit HIV-1 replication (109, 110).

This “phase out” concept is further supported by the natural hosts of SIV (i.e., non-pathogenic infection); African green monkeys and sooty mangabeys, that demonstrate a decrease in the expression of ISGs and systemic activation just weeks after SIV infection (111, 112). These natural hosts of SIV also differ from HIV infection in humans (as well as pathogenic SIV infection in rhesus macaque monkeys) in that they have a lack of microbial translocation from the gut and very few memory CD4^+^ T cells are infected [reviewed in Ref. (113)]. Therefore, the IFN-I response in humans is most likely also beneficial in the early stages of infection and would be of greater benefit if it remained confined to mucosal barriers and viral reservoirs. However, if the infection is never cleared, inflammation becomes systemic and the ongoing production of IFN-Is becomes detrimental to the host (i.e., human) in the chronic phase.

Regarding this issue, Dallari et al. identified two SRC family kinases, FYN and LYN, that were constitutively activated in pDCs, potentially providing a useful target, as pDCs are deemed to be the most important IFN-I-producing cell in chronic infection (114). But are there other producer cells that also need to be targeted? And even if pDCs do turn out to be the primary producers during chronic infection, their scarcity and distribution in tissues makes them to difficult to access for *ex vivo* analyses. There is a real possibility that different cell subset(s) are producing IFN-I after peak viral load has been reached. It is also still unclear if pDCs are producing too much or too little IFN-I in HIV-1 patients in chronic infection. Certainly pDCs decrease from acute to chronic infection (in the non-pathogenic models of SIV) (115–118). It is also known that pDCs migrate from the blood to draining lymph nodes before apoptosis (119, 120), however, it is still unknown if the pDCs that migrate from the blood to the rectum or vagina continue to produce IFN-Is or also undergo apoptosis.

In addition to pDCs, DCs and macrophages potently produce IFN-Is after HIV-1 infection as described above. These cells reside at common sites of infection, such as the vagina and rectum (121, 122) and IFN-I expression is increased at these sites after SIV infection in rhesus macaques (123). Therefore, it is likely that not only pDCs but also myeloid cells such as DCs and macrophages contribute to IFN-I secretion at the port of viral entry (e.g., vaginal and rectal tissues).

To further reveal the significance of IFN-I responses in pathogenic HIV/SIV infection *in vivo*, Sandler et al. showed that by blocking IFNAR using an IFN-I antagonist immediately after SIV infection in rhesus macaque monkeys, SIV reservoir size was increased, anti-viral gene expression was decreased, and CD4^+^ T cell depletion was accelerated leading to a progression to AIDS (124). This study highlighted the importance of IFN-I responses and how crucial they are for control of SIV infection in the acute phase. Additionally in this study, IFN-α2a, a subtype of IFN-I, was administered from 1 week prior to infection in a different cohort of macaque monkeys resulting in the initial upregulation of ISGs and prevention of systemic infection (124). However, prolonged administration resulted in IFN-I desensitization, decreased anti-viral ISG expression, increased SIV reservoir size, and the loss of CD4^+^ T cell loss (124), suggesting IFN-I somewhat has the properties of a double-edged sword for/against pathogenic HIV/SIV infection *in vivo*.

To directly elucidate the impact of IFN-I in HIV-1 infection *in vivo*, certain groups have utilized HSC-transplanted humanized mouse models. First, Zhen et al. showed that in a humanized mouse model of chronic HIV-1 infection, blocking IFNAR in combination with cART could accelerate viral suppression, reduce the viral reservoir, and further decrease T cell exhaustion and HIV-1-driven immune activation while also restoring HIV-1-specific CD8^+^ T cell functions (125). Secondly, because the IFN-I response perseveres even under cART, Cheng et al. attempted to combine IFNAR blockade with cART, showing a reduction in the HIV-1 reservoir in lymphoid tissues, demonstrated by a delay in viral replication rebound following cART cessation (126). Thirdly, in another study by Cheng et al., IFNAR was blocked from weeks 6–10 post-infection (i.e., the chronic phase of infection) (127) resulting in increased viral replication correlating with elevated T cell activation, suggesting that IFN-Is suppress HIV-1 replication during the chronic phase but are not essential for HIV-1-induced aberrant immune activation (127). This study demonstrated that persistent IFN-I signaling during the chronic phase of infection may help to dampen HIV-1 viral replication although it also contributes to the depletion of CD4^+^ T cells (127).

## Future Direction

Here, we have described the positive and negative aspects of IFN-I responses once HIV-1 infection has occurred. Based on *in vitro* investigations using cell cultures, IFN-I quite efficiently suppresses HIV-1 replication (presumably inducing robust RFs) (Figure 3). In sharp contrast, the effect of IFN-I in the *in vivo* environment seems much more complicated than expected from previous knowledge around *in vitro* analyses using cell cultures. Future deep and comprehensive investigations using animal models, particularly monkey models for SIV infection and humanized mouse models for HIV-1 infection, will be important to shed light on the true behavior of IFN-I for/against viral infections.

## Author Contributions

KS conceived the outline of the manuscript; all authors contributed to writing the manuscript.

## Conflict of Interest Statement

The authors declare that the research was conducted in the absence of any commercial or financial relationships that could be construed as a potential conflict of interest.
